# Multilocus Phylogenetic Study of the *Scheffersomyces* Yeast Clade and Characterization of the N-Terminal Region of Xylose Reductase Gene

**DOI:** 10.1371/journal.pone.0039128

**Published:** 2012-06-14

**Authors:** Hector Urbina, Meredith Blackwell

**Affiliations:** Department of Biological Sciences, Louisiana State University, Baton Rouge, Louisiana, United States of America; Louisiana State University, United States of America

## Abstract

Many of the known xylose-fermenting (X-F) yeasts are placed in the *Scheffersomyces* clade, a group of ascomycete yeasts that have been isolated from plant tissues and in association with lignicolous insects. We formally recognize fourteen species in this clade based on a maximum likelihood (ML) phylogenetic analysis using a multilocus dataset. This clade is divided into three subclades, each of which exhibits the biochemical ability to ferment cellobiose or xylose. New combinations are made for seven species of *Candida* in the clade, and three X-F taxa associated with rotted hardwood are described: *Scheffersomyces illinoinensis* (type strain NRRL Y-48827^T^  =  CBS 12624), *Scheffersomyces quercinus* (type strain NRRL Y-48825^T^  =  CBS 12625), and *Scheffersomyces virginianus* (type strain NRRL Y-48822^T^  =  CBS 12626). The new X-F species are distinctive based on their position in the multilocus phylogenetic analysis and biochemical and morphological characters. The molecular characterization of xylose reductase (XR) indicates that the regions surrounding the conserved domain contain mutations that may enhance the performance of the enzyme in X-F yeasts. The phylogenetic reconstruction using *XYL1* or *RPB1* was identical to the multilocus analysis, and these loci have potential for rapid identification of cryptic species in this clade.

## Introduction

D-xylose is a five-carbon backbone molecule of the hemicellulose component of plant cell walls and is one of the most abundant renewable carbon resources on Earth. Some bacteria and certain fungi, including fewer than twenty-five species of the more than 1500 described ascomycete yeasts, share the ability to produce ethanol by the fermentation of D-xylose [1]. In order to ferment D-xylose, yeasts express xylose reductase (XR), xylitol dehydrogenase (XDH), and xylulose kinase (XK) to convert D-xylose to D-xylulose-5-phosphate; D-xylulose-5-phosphate is then incorporated into the pentose phosphate pathway to be catalyzed to ethanol [2]–[4].

Xylose fermentation has been the focus of several studies in order to identify differences in the catabolic rate between strains [2], [5]-[14]. Overexpression, homologous and heterologous expression, and direct mutagenesis of genes involved in D-xylose assimilation and fermentation have only modestly enhanced the quantity of ethanol production by yeasts due to several metabolic constraints; these include rate of regeneration of the cofactor NADP(H) required by XR and XDH, repression by glucose, and anaerobic respiration regulatory control [15]-[17]. These studies have resulted in the present understanding of the biochemical pathway, but the main goal of bioengineering yeasts capable of fermenting D-xylose at a high rate to be used at industrial scales has not yet been achieved. Consequently, recent research has been focused on the discovery of new X-F yeasts, e.g. *Spathaspora passalidarum* and *Candida jeffriesii* [18], and *Spathaspora arborariae* and other taxa [19], [20], from the guts of lignicolous beetles and rotted wood, niches from which a number of X-F yeasts have been isolated [21]-[23].

Although, X-F yeasts appear scattered throughout the Saccharomycotina, the yeasts that have been reported to exhibit the highest rate of xylose fermentation under certain conditions are members of the *Scheffersomyces* clade [9], [21], [24], and for this reason fermentative ability has been intensively studied in this clade [9], [25]-[51]. Few studies, however, have undertaken clarification of the phylogenetic relationships among the yeasts of the clade [1], [52]-[58]. Therefore, a robust, well-supported phylogeny including as many taxa as possible is necessary to clarify the phylogenetic relationships among the *Scheffersomyces* clade members along with a comparison of the nucleotide mutations and enzymatic activity of the XR to understand the importance of the biochemical ability in the speciation process of this yeast clade.

**Figure 1:**
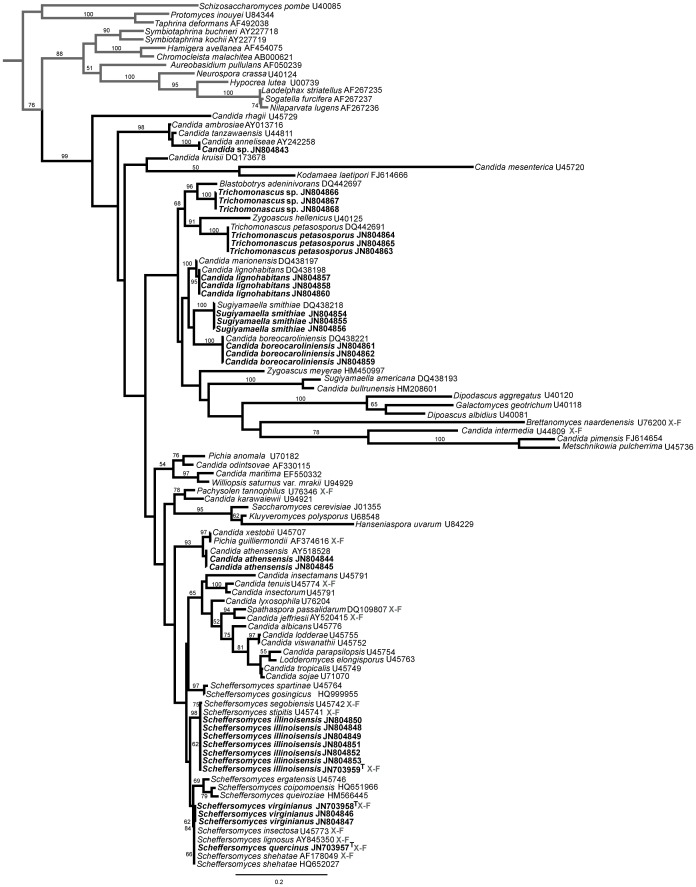
ML tree based of the D1/D2 LSU region using a 606-character matrix for yeast species isolated from the wood samples (in bold). *Schizosaccharomyces pombe* was used as an outgroup taxon (in grey). X-F, xylose-fermenting yeasts. Numbers above each branch refer to bootstrap values out of 1000 repetitions. ML score -11353.90.

In order to distinguish the species in the *Scheffersomyces* clade we used BLAST searches, biochemical and morphological characterization, and a multilocus phylogenetic analysis that included the traditional SSU and LSU markers, the orthologous *RPB1*, and the recently proposed ITS barcoding region for fungi [59]. We present a taxonomic revision of the *Scheffersomyces* clade, trace the nucleotide differences in the *XYL1*, and propose three new species of X-F yeasts, *Scheffersomyces illinoinensis, Scheffersomyces quercinus*, and *Scheffersomyces virginianus* associated with rotted hardwoods, *Carya illinoinensis* (pecan), *Quercus nigra* (water oak), and *Quercus virginiana* (live oak). We performed a molecular study of the *XYL1* that codifies XR in certain members of the *Scheffersomyces* clade.

**Table 1:** GenBank accession numbers of the nucleotide sequences used in this study. Sequences generated in this work shown in bold.

Species	Codes	SSU	ITS	LSU	*RPB1*	*XYL1*
*C. bolitotheri* ^T^	NRRL Y-27587, CBS 9832, BG 00-8-15-1-1	AY242142	FJ623599	AY242249	**JN804828**	-
*C. terraborum* ^T^	NRRL Y-27573, CBS 9826, BG 02-7-15-019A-2-1	AY426956	FJ623596	AY309810	**JN804831**	-
*C. panamericana* ^T^	NRRL Y-27567, CBS 9834, BG 01-7-26-006B-2-1	AY426960	FJ623601	AY309872	**JN804835**	-
*S. coipomoensis* ^T^	NRRL Y-17651, ATCC 58904, CBS 8178	HQ651931	HQ652070	HQ651966	EU344070	-
*S. lignicola* ^T^	ATCC MYA-4674, CBS 10610, BCC 7733	AY845351	HQ652074	AY845350	-	-
*S. ergatensis* ^T^	NRRL Y-17652, ATCC 22589, CBS 6248	AB013524	EU343826	U45746	EU344098	**JQ436926**
*S. insectosa* ^T^	NRRL Y-12854, ATCC 66611, CBS 4286	AB013583	HQ652064	FM200041	**JN804842**	**JQ235697**
*S. lignosus* ^T^	NRRL Y-12856, ATCC 58779, CBS 4705	HQ651941	**JN943262**	U45772	**JN804837**	**JQ235693**
*S. segobiensis* ^T^	NRRL Y-11571, ATCC 58375, CBS 6857	AB054288	DQ409166	U45742	EF599429	**JQ436925**
*L. elongisporus* ^T^	NRRL YB-4239, ATCC 11503, CBS 2605	HQ876033	HQ876042	HQ876050	AY653537	-
*C. tropicalis* ^T^	NRRL Y-12968, ATCC 4563, CBS 616	EU348785	AB437068	U45749	-	-
*S. queiroziae* ^T^	NRRL Y-48722, UFMG-CLM 5.1, CBS 11853	-	HM566445	HM566445	-	-
*S. gosingicus* ^T^	CBS-11433, BCRC 23194, SJ7S11	HQ876040	HQ999978	HQ999955	-	-
*S. spartinae* ^T^	NRRL Y-7322, ATCC 18866, CBS 6059	FJ153139	HQ876044	U45764	-	-
*S. stipitis* ^T^	NRRL Y-7124, ATCC 58376, CBS 5773	AB054280	**JN943257**	U45741	**JN804841**	**JQ235696**
*Scheffersomyces* sp.	NRRL Y-48762, CBS 12363, UFMG HMD-26.3	-	JF826438	JF826438	-	-
*S. shehatae* ^T^	NRRL Y-12858, CBS 5813, ATCC 34887	AB013582	**JN943264**	AF178049	**JQ436927**	**JQ235691**
***S. quercinus*** **^T^**	**NRRL Y-48825T, CBS** **12625, W07-09-15-1-3-2**	**JN940981**	**JN943260**	**JN703957**	**JN804838**	**JQ008829**
***S. virginianus*** **^T^**	**NRRL Y-48822T, CBS** **12626, W07-10-04-4-6-2**	**JN940969**	**JN943259**	**JN703958**	**JN804839**	**JQ235695**
***S. illinoinensis*** **^T^**	**NRRL Y-48827T, CBS** **12624, W07-11-15-9-2-1**	**JN940968**	**JN943261**	**JN703959**	**JN804840**	**JQ235694**

**Table 2:** Nucleotide differences and percentages of homology between the new xylose-fermenting yeasts and the type cultures of closest relatives, *S. shehatae* or *S. stipitis*.

Species	SSU	ITS	D1/D2 LSU	*RPB1*	*XYL1*
*S. insectosa* ^T^	100%	98% (5 n)	100%	94% (34 n)	92% (44 n)
*S. lignosus* ^T^	100%	98% (5 n)	100%	94% (37 n)	91% (46 n)
***S. quercinus*** **^T^**	98% (27n)	99% (1 n)	98% (7 n)	98% (10 n)	96% (11 n)
***S. virginianus*** **^T^**	97% (54n)	99% (1 n)	99% (4 n)	98% (10 n)	97% (8 n)
***S. illinoinensis*** **^T^**	99% (3n)	99% (3 n)	100%	95% (28 n)	97% (15 n)

**Table 3:** Differences in physiological reactions of *S. quercinus, S. illinoinensis*, and *S. virginianus* and their closest relatives*.

Species	Rhammose	Galactitol	Lactate	Cadaverine	Glucosamine	Tryptophan	Melibiose	Inulin
*S. insectosa*	-	-	-	+	-	-	-	-
*S. lignosus*	-	+	w	+	-	-	-	-
***S. quercinus***	-	+,**f**	+	+	+	+	-	+
*S. shehatae*	-	-	-	+	-	-	-	-
***S. virginianus***	**w**	**w**	-	-	+	+	+	+
*S. stipitis*	+	-	+/−	+	-	-	-	-
***S. illinoinensis***	+	+/**d**	+/**d**	**w**	+	+	**w**	**w**

* Biochemical assay results of previously described species were complied from Kurtzman (1990) and Barnett et al. (2000). Abbreviations for reaction results: +, positive; -, negative; d, delayed positive; w, weak positive.

## Materials and Methods

### Yeast Isolation and Culture

Partially decayed logs and fallen branches of *Carya illinoinensis* (pecan, 30 cm diam), *Quercus nigra* (water oak, approximately 10 cm diam), and *Quercus virginiana* (live oak, approximately 10 cm diam) were collected from Pecan Drive, Saint Gabriel, Ascension Parish, Louisiana, and LSU Burden Center and the corner of Highland Road and S. Stadium Drive on the LSU campus, Baton Rouge, East Baton Rouge, Louisiana, respectively, between Sep and Oct 2007. The wood samples were divided into approximately 1 cm^2^ samples, and each sample was placed in a 1.5 mL microcentrifuge tube with 1 mL of sterile water. Tubes were vortexed for 30 s and a 100 µL aliquot was plated on acidified yeast medium agar [60]. Plates were incubated for 3 d at 25 C, and single colonies were isolated and streaked 4 times to obtain pure cultures.

### Molecular Identification

Genomic DNA was extracted using a Wizard® Genomic DNA purification kit (Promega). The concentration, integrity, and purity of total DNA extracted were confirmed by gel electrophoresis in 0.8% agarose in 0.5 × Tris-Borate-EDTA (TBE) buffer. Initial rapid identification was carried out by PCR amplification and sequencing of the LSU (D1/D2 region ∼600 bp) rRNA gene for use in BLAST searches [1], [54]. In order to increase the robustness of the phylogenetic analyses, PCR amplifications of the small subunit (SSU ∼1.6 Kbp) and internal transcribed spacers 1 and 2 (ITS ∼500 bp) of the rRNA marker were carried out in addition to the D1/D2 region [61], [62] and the SSU rRNA gene was amplified using the combination of primers NS1 (forward) (5′-GTAGTCATATGCTTGTCTC-3′) and NS8 (reverse) (5′-TCCGCAGGTTCACCTACGGA-3′); ITS1-LSU markers were amplified using the combination of primers ITS1 (forward) (5′-TCCGTAGGTGAACCTGCGG-3′) and LR3 (reverse) (5′-CCGTGTTTCAAGACGGG-3′) in a PCR reaction with 20 µg of total DNA, 0.5 mM DTPs, 2.5 mM MgSO4, 0.8 µM of each primer set, 1 × PCR buffer, and 1 U of Taq polymerase (Promega) in 25 µL of final volume. The PCR amplification protocol included 5 min of DNA pre-denaturation at 95 C followed by 35 cycles of 1 min of DNA denaturation at 95 C, 45 s of primer annealing at 55 C, and a 2 min extension at 72 C, and 10 min final PCR extension.

**Figure 2:**
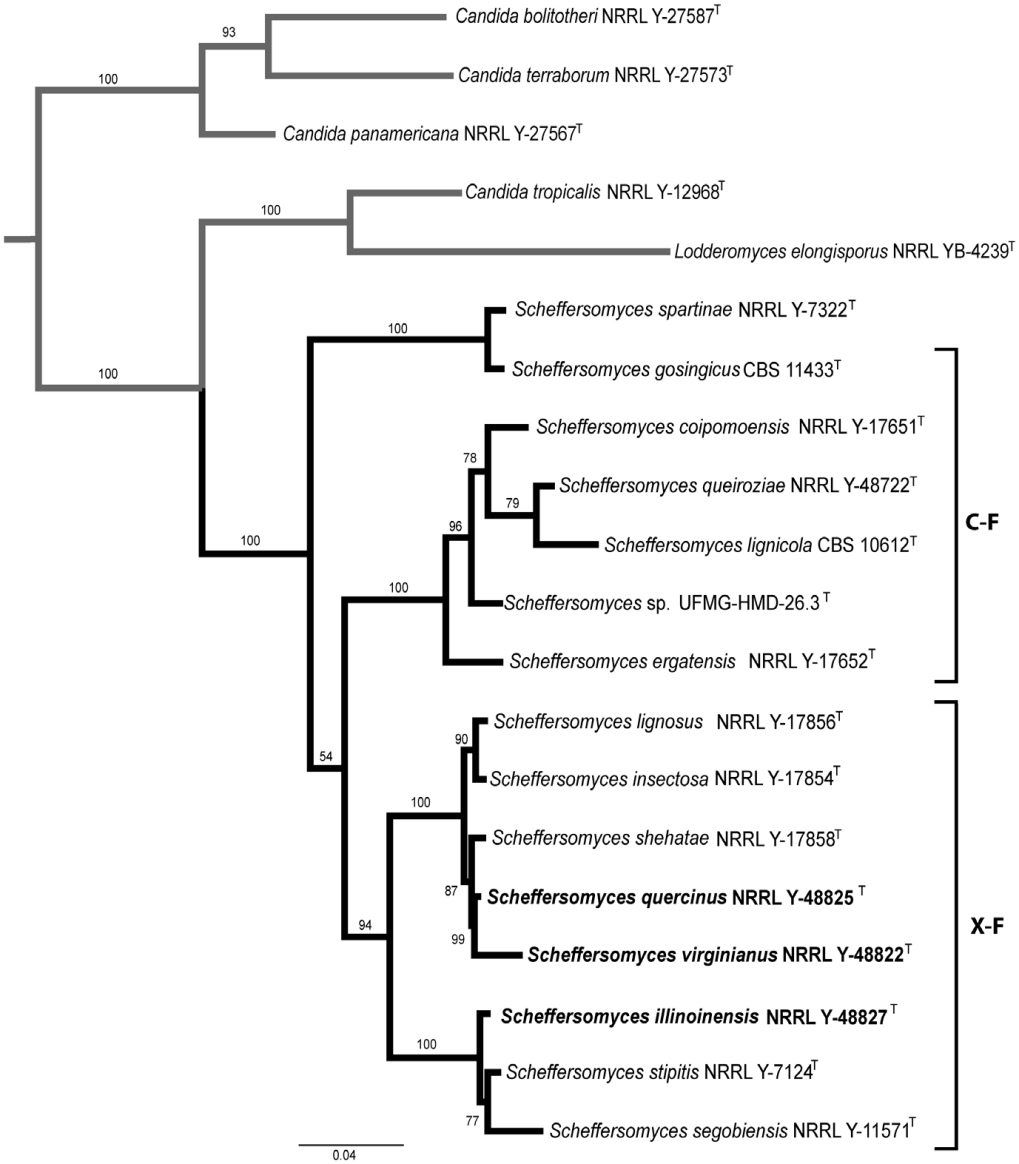
ML tree based on a multilocus dataset using a 3488-character matrix for *Scheffersomyces* clade. *Candida tropicalis* was used as an outgroup taxon (in grey). C-F and X-F, cellobiose- and xylose-fermenting yeasts respectively. Numbers above each branch refer to bootstrap values out of 1000 repetitions. ML score is -13300.52.

**Figure 3:**
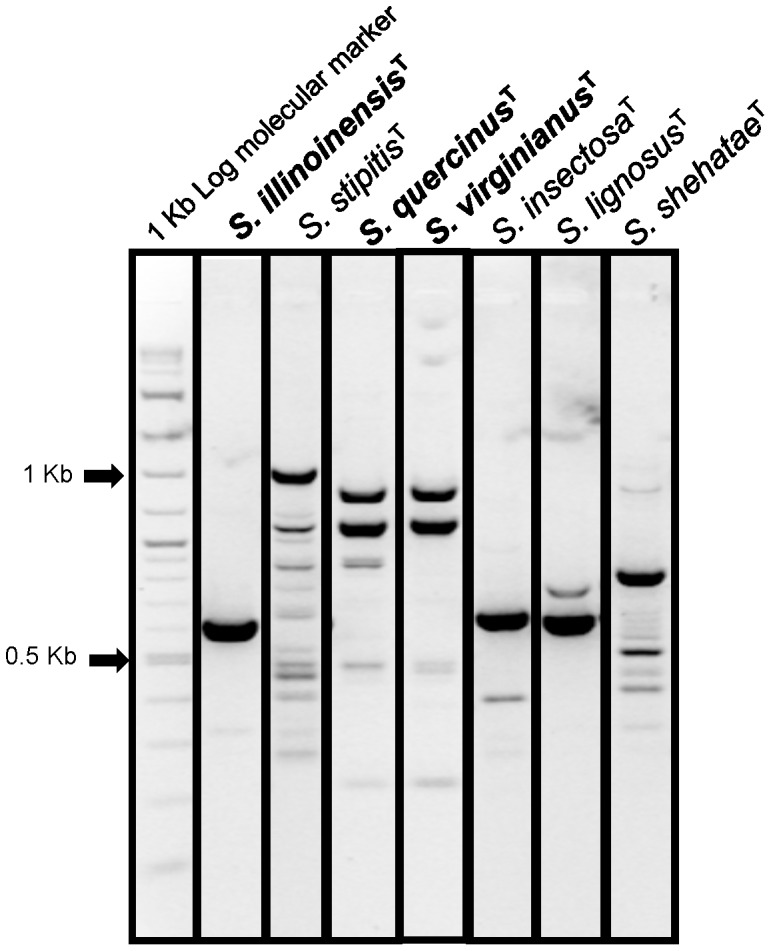
Characterization of *S. quercinus, S. illinoinensis*, and *S. virginianus* using RAPID-PCR CDU fingerprinting primers.

In addition, another nuclear locus, RNA polymerase II, was used in the phylogenetic analysis. A fragment of ∼700 bp of the subunit I (*RPB1*) gene was amplified by the primer pair RPB1-Af (forward) 5′-GARTGYCCDGGDCAYTTYGG-3′ and RPB1-Cr (reverse) 5′-CCNGCDATNTCRTTRTCCATRTA-3′ [63], [64]. The PCR reaction was performed using 100 µg of total DNA, 0.6 mM DTPs, 2.5 mM MgSO4, 1 µM of each primer, 1 × PCR buffer, and 1.5 U of Taq polymerase (Promega) in 35 µL total final volume of reaction. The PCR amplification program included 5 min of DNA pre-denaturation at 95 C followed by 35 cycles of 1 min of DNA denaturation at 95 C, 45 s of primer annealing at 55 C, and 2 min of extension at 72 C, and 10 min final PCR extension.

The purified PCR products were sequenced in both directions by Beckman Coulter Genomics (Danvers, MA). Each molecular marker was sequenced on three independent occasions in order to avoid nucleotide differences due to sequencing errors.

**Figure 4:**
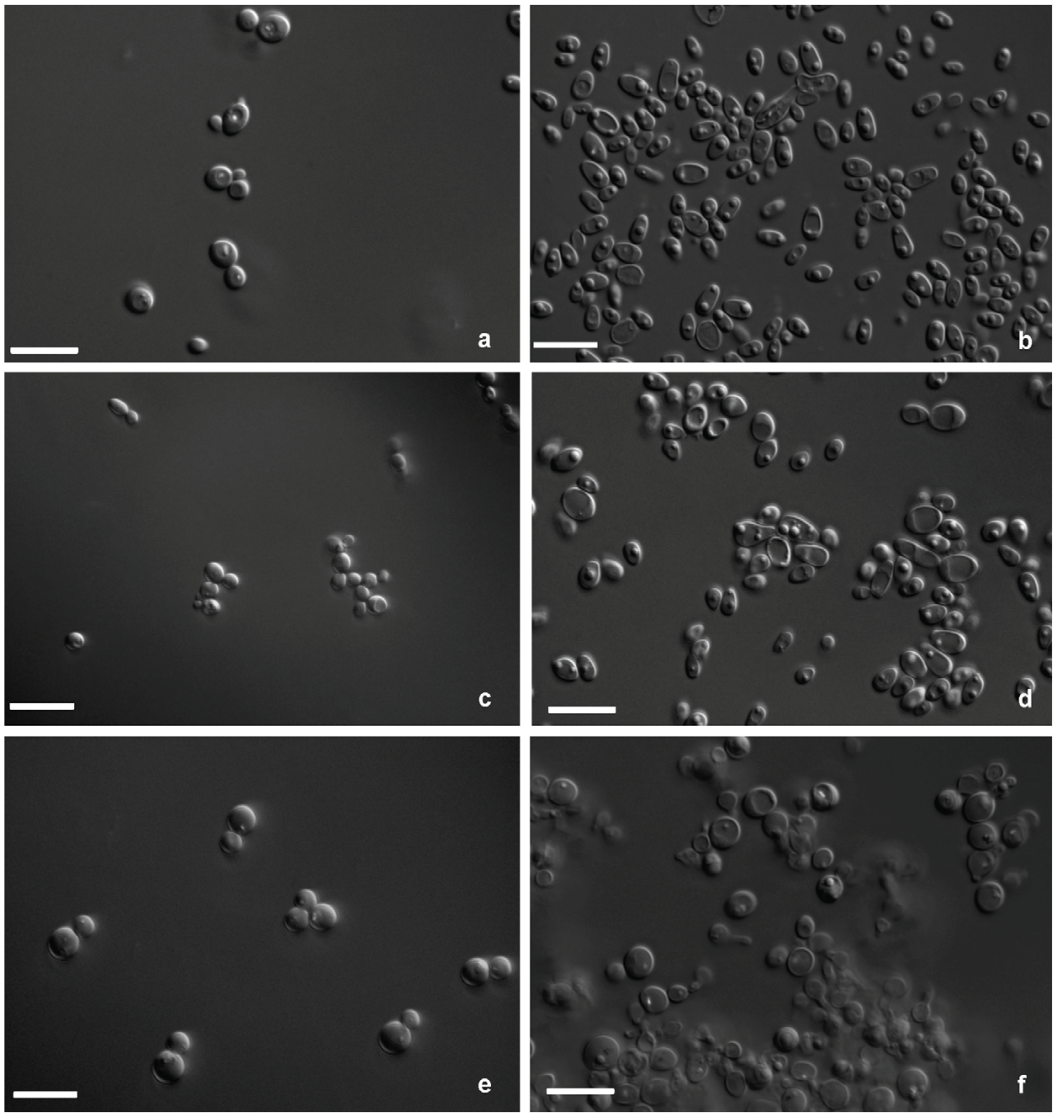
Morphological characterization of *S. quercinus*, budding cells (a-b); *S. virginianus,* budding cells (c-d); and *S. illinoinensis*, budding cells (e-f); grown at 25 C in YM broth and V8 agar at 7 d days respectively, bar 10 µm.

### RAPD-PCR Fingerprinting

RAPD–PCR analysis was performed with the oligonucleotide primer CDU (5′-GCGATCCCCA-3′) [65]-[68]. Aliquots of 25 µL of amplified product were analyzed by electrophoresis on 1.8% agarose gel in 1× TBE buffer with 1 × SYBR® Safe DNA Gel Stain (Invitrogen, Grand Island, NY) at 70 V for 80 min. DNA fragments were visualized with a UV-light transilluminator and photographed using a Polaroid system.

### Morphological, Biochemical and Physiological Characteristics

The yeast standard description based on phenotypic characters was executed following standardized protocols [1], [69], [70].

### Xylose Reductase (XR) Molecular Studies

The ∼600 bp fragment of the *XYL1* was amplified using the following degenerate primers: XYL1-forward (5′-GGTYTTYGGMTGYTGGAARSTC-3′) and XYL1-reverse (5′-AAWGATTGWGGWCCRAAWGAWGA-3′) designed in this study, in a PCR reaction with 100 µg of total DNA, 0.4 mM DTPs, 4 mM MgSO_4_, 1 µM of each primer, 1× PCR buffer, and 1U of Taq polymerase (Promega) in a final volume of 25 µL. The PCR amplification program included 5 min of DNA pre-denaturization at 95 C followed by 35 cycles of 1 min of denaturization at 95 C, 1 min primer annealing at 57 C, 1 min extension at 72 C, and 10 min final PCR extension. The purified PCR products were sequenced as described above.

### Phylogenetic Analyses

Contig sequences and sequencing manipulations were performed with Se-AL v2.01a11 (http://tree.bio.ed.ac.uk/software/seal/) and MESQUITE v2.74 [71]. The sequence alignments were carried out using the online interface MAFFT v6.859 (http://mafft.cbrc.jp/alignment/software/) with different advanced alignment strategies per locus: LSU and *RPB1*, global homology (G-INS-i); ITS, one conserved domain (L-INS-i); and SSU, secondary structure of RNA (Q-INS-i). In particular ITS loci were realigned using the software SATé v2.1.2 [72], and ambiguous sequence alignment ends were eliminated in all the alignments. Maximum likelihood (ML) phylogenic inference was performed in RAxML-VI-HPC [73] using a partitioned multilocus matrix (each partition for SSU, ITS1, 5.8 S, ITS2, LSU, and *RPB1*) under a general time reversible model with a gamma distribution of site rate variation (GTRGAMMA), and ML support was estimated using 1000 bootstrap replicates. The sequences for RPB1 and XR were obtained using SEQUIN v11.0 (http://www.ncbi.nlm.nih.gov/Sequin/) with the alternative yeast nuclear codon bias [45], [74]. Phylogenetic analysis using the amino acid matrix was performed in RAxML-VI-HPC under the VT AA selection model [75] and ML support was estimated using 1000 bootstrap replicates. Tree editing was done with FigTree v1.3.1 software (http://tree.bio.ed.ac.uk/software/figtree/).

**Table 4:** Biochemical characterization of S. quercinus, S. illinoinensis, and S. virginianus.

		*S. quercinus*	*S. illinoinensis*	*S. virginianus*
	**Fermentation**			
F1	D-Glucose	+	+	+, f
F2	D-Galactose	+	+	+
F3	Maltose	w	+	+
F4	α-Methyl-D-glucoside	-	-	-
F5	Sucrose	-	-	-
F6	α,α- Trehalose	+	+	+
F7	Melibose	-	w	-
F8	Lactose	-	-	-
F9	Cellobiose	-	-	-
F10	Melezitose	-	-	-
F11	Raffinose	-	-	-
F12	Inulin	-	-	-
F13	Starch	-	-	-
F14	D-Xylose	+	+	+
	**Assimilation**			
C1	D-Glucose	+, f	+, f	+
C2	D-Galactose	+, f	+, f	+, f
C3	L-Sorbose	+,d	-	w
C4	D-Glucosamine	w	-	+
C5	D-Ribose	+,d	+,d	+, d
C6	D-Xylose	+	+	+
C7	L-Arabinose	+,d	+	+
C8	D-Arabinose	w	+	w
C9	L-Rhamnose	-	+	w
C10	Sucrose	+	+	+
C11	Maltose	+	+, f	+
C12	Trehalose	+	+, f	+
C13	α-Methyl-D-glucoside	+	+	+
C14	Cellobiose	+	+	+
C15	Salicin	+	+	+
C16	Arbutin	+	+	+
C17	Melibiose	-	+	+
C18	Lactose	-	-	+
C19	Raffinose	-	-	w
C20	Melezitose	-	+	+, d
C21	Inulin	w	w	+
C22	Soluble Starch	+	+	+
C23	Glycerol	+	+	+
C24	Erythritol	+	+	+
C25	Ribitol	+	+	+
C26	Xylitol	+, d	+	w
C27	L-Arabinitol	+, d	+,d	-
C28	D-Glucitol	+	+	+
C29	D-Mannitol	+	+	+
C30	Galactitiol	+, f	+, f	w
C31	myo-Inositol	-	-	w
C32	D-Glucono-1,5-lactone	+	+	+
C33	2-Keto-D-gluconate	+	+,f	+, f
C34	5-Keto-D-gluconate	?	?	?
C35	D-Gluconate	+	+,d	+
C36	D-Glucuronate	-	-	-
C38	DL-Lactate	+	+,d	-
C39	Succinate	+	+	+
C40	Citrate	+, d	+	+
C41	Methanol	-	-	-
C42	Ethanol	+, f	+,f	+, f
C43	Propane 1,2 diol	+, d	w	w
C44	Butane 2,3 diol	w	-	w
C45	Quinic acid	-	-	w
C46	D-Glucarate	-	-	-
	**Temperature**			
T1	30°C	+	+	+
T2	35°C	+, f	+,f	+
T3	37°C	-	+,d	-
	**Osmotic pressure**			
O1	0.01% Cycloheximide	+, f	+, f	+, f
O2	0.1% Cycloheximide	+, f	+, f	+,f
O3	1% Acetic Acid	-	-	-
O4	50% D-Glucose	+	+	+
O5	60% D-Glucose	w	+	w
O6	10% NaCl	+	+	w
O7	16% NaCl	-	-	-
	**Nitrogen assimilation**			
N1	Nitrate	-	+, d	+
N2	Nitrite	-	+, d	-
N3	Ethylamine	+	+	+
N4	L-Lysine	+, d	-	+
N5	Cadaverine	+	w	-
N6	Creatine	-	-	-
N7	Creatinine	-	-	-
N8	D-Glucosamine	+	+	+
N9	Imidazole	-	-	-
N10	D-Tryptophan	+	+	+
	**Vitamins**			
V1	w/o Vitamins	-	-	-
V2	w/o myo-Inositiol	+, d	w	+
V3	w/o Pantothenate	+	+	+
V4	w/o Biotin	+	-	-
V5	w/o Thiamin	+	+	+
V6	w/o Biotin & Thiamin	-	-	w
V7	w/o Pyridoxine	+	+	+
V8	w/o Pyrid. &Thiam	+	+	+
V9	w/o Niacin	+	+	+
V10	w/o PABA	+	+	+

Abbreviations for reaction results: +, positive; -, negative; d, delayed positive; w, weak positive.

## Results

### Diversity of Yeasts Isolated from Rotted Wood

The initial rapid molecular identification of the 29 yeast strains isolated from the wood samples using the D1/D2 LSU region confirmed the presence of several closely related species classified in the *Scheffersomyces* (11 isolates), *Sugiyamaella* (10 isolates), *Trichomonascus* (5 isolates), *Meyerozyma* (2 isolates), and *Candida tanzawaensis* (1 isolate) clades (Fig. 1). Initial biochemical characterization of fermentation abilities performed on all the isolates showed that only strains classified in the *Scheffersomyces* clade had the ability to ferment xylose.

**Figure 5:**
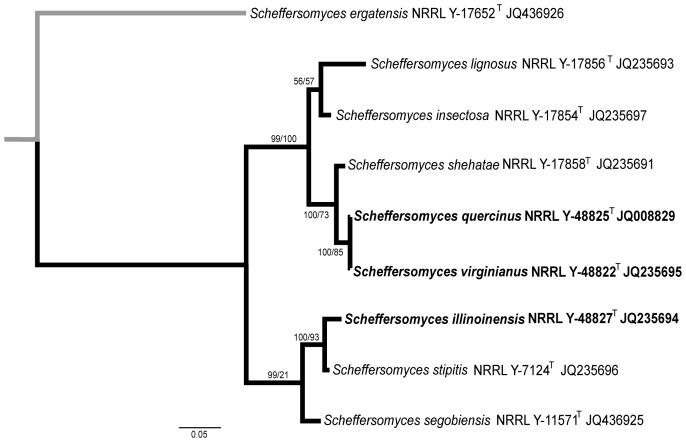
ML consensus tree based on *XYL1* and the putative XR from of X-F members in *S. stipitis* subclade. *Scheffersomyces ergatensis* was used as an outgroup taxon (in grey). Numbers above each branch refer to bootstrap values out of 1000 repetitions. ML scores are -1829.90 (DNA data) and -1284.79 (amino acid data).

**Figure 6:**
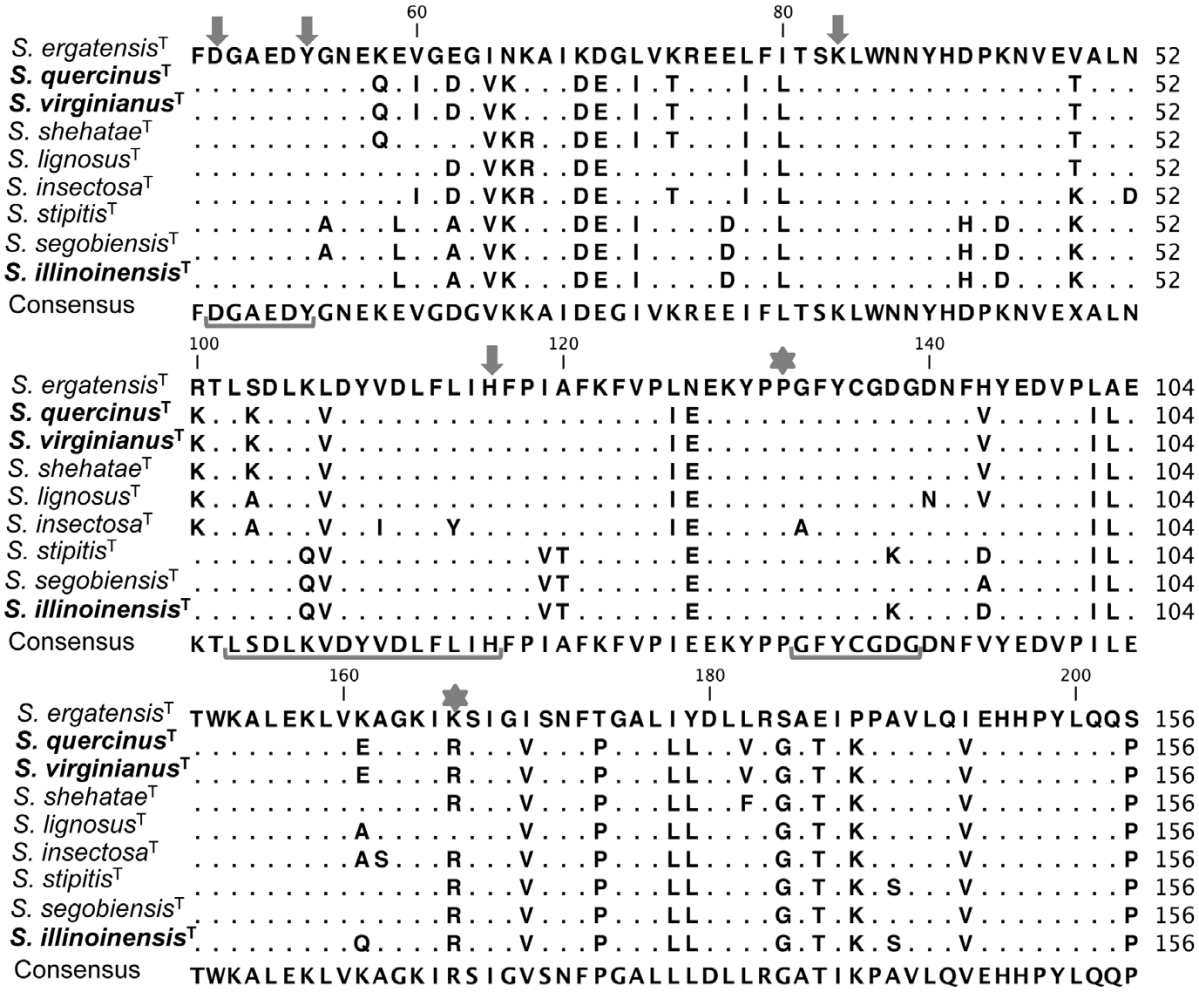
Multiple sequence alignment of the N-terminal region of XR. Identical residues are indicated by dots. Arrows indicate amino acids that constitute the active sites, stars indicate amino acids that form the xylose-binding pocket, and brackets indicate conserved domains.

### New Xylose-fermenting Yeasts

Species delimitation of *S. quercinus, S. virginianus,* and *S. illinoinensis,* was based on phylogenetic placement, nucleotide differences in the rRNA markers (SSU, ITS and LSU), *RPB1* and *XYL1*, and biochemical assay differences compared with their closest relatives (Table 1, 2 and 3, Fig. 2). In addition, we obtained different banding patterns using CDU RAPID-PCR fingerprinting primers (Fig. 3). These fingerprinting primers have been used previously to discriminate among cryptic yeast species [66], [67], [76].

### Species Description of *Scheffersomyces quercinus* H. Urbina & M. Blackw. sp. nov

MycoBank accession number MB563719, (Fig. 4, a-b).

After 7 d growth in YM broth at 25 C, cells are subglobose (5–8×5–7.5 µm), and occur singly, in pairs, or in chains. Pseudohyphae are present; true hyphae are absent. After 7 days on YM agar at 25 C, colonies are cream-colored with pale-pinkish perimeter on some older colonies, smooth, flat, and/or with scattered filaments at the margin. After 10 d of Dalmau plate culture on corn meal agar at 25 C, true hyphae are present. Aerobic growth is white, shiny, and smooth with filamentous margin. Asci and ascospores are not observed on YM or V8 agar. Diazonium blue B reaction is negative. See Table 4 for physiological characterization.

#### Type strain

NRRL Y-48825^T^ ( = CBS 12625; W07-09-15-1-3-2) is preserved as a lyophilized preparation in the Agricultural Research Service Culture Collection (NRRL), Peoria, Illinois, USA. The strain was isolated from rotted wood (*Quercus niger*), collected on 15 Nov 2007 at Louisiana State University, Burden Research Plantation, Baton Rouge, East Baton Rouge Parish, Louisiana, USA, N30**°**40′59″-W91**°**10′29″.

#### Etymology

The species name *quercinus* (N.L. gen. n.) refers to the genus of the substrate, *Quercus niger*, from which this species was isolated.

### Species Description of *Scheffersomyces virginianus* H. Urbina & M. Blackw. sp. nov

MycoBank accession number MB 563720 (Fig. 4, c-d).

After 7 d growth in YM broth at 25 C, cells are globose to ellipsoidal (5.5–10×4–6.5 µm), and occur singly, in pairs, in short chains, or in small clusters. Pseudohyphae are present; true hyphae are absent. After 7 d on YM agar at 25 C, cream-colored to light pink with abundant filaments at margin. After 10 d Dalmau plate culture on corn meal agar at 25 C, pseudohyphae are present; septate hyphae are absent. Aerobic growth is white, shiny, and smooth with filaments at margin. Asci and ascospores are not observed on YM or V8 agar. Diazonium blue B reaction is negative. See Table 4 for physiological characterization.

### Type Strain

NRRL Y-48822^T^ ( = CBS 12626; W07-10-04-4-6-2) is preserved as a lyophilized preparation in the Agricultural Research Service Culture Collection (NRRL), Peoria, Illinois, USA. The strain was isolated from rotted wood (*Quercus virginiana*) collected on 15 Sep 2007, Highland Rd. and S. Stadium Dr., Louisiana State University, Baton Rouge, East Baton Rouge Parish, Louisiana, USA, N30**°**40′90″-W91**°**17′60″.

### Etymology

The species name *virginianus* (N.L. gen. n.) refers to the species of the substrate, *Quercus virginiana*, from which this species was isolated.

### Species Description of *Scheffersomyces illinoinensis* H. Urbina & M. Blackw. sp. nov

MycoBank accession number MB563457 (Fig. 4, e-f).

After 7 d growth in YM broth at 25 C, cells are globose (5−7.5 µm), and occur singly, in pairs, or in short chains. Pseudohyphae and hyphae are not present. After 7 d on YM agar at 25 C, colonies are cream-colored, smooth, and flat with a smooth margin. After 10 d Dalmau plate culture on corn meal agar at 25 C, pseudohyphae are present; septate hyphae are absent. Asci and ascospores are not observed on YM and V8 agar. Diazonium blue B reaction is negative. See Table 4 for physiological characterization.

#### Type strain

NRRL Y-48827^T^ ( = CBS 12624; W07-11-15-9-2-1) is preserved as a lyophilized preparation in the Agricultural Research Service Culture Collection (NRRL), Peoria, Illinois, USA. The strain was isolated from rotted wood (*Carya illinoinensis*) collected on 15 Nov 2007 at 810 Pecan Dr., St. Gabriel, Ascension Parish, Louisiana, USA, N30**°**30′56″-W91**°**10′30″.

#### Etymology

The species name *illinoinensis* (N.L. gen. n.) refers to the species of the substrate, *Carya illinoinensis*, from which this species was isolated.

### Multilocus Phylogenetic Study

The phylogenetic placement of *S. quercinus, S. virginianus*, and *S. illinoinensis* was based on ML analysis results of a concatenated nucleotide dataset containing 3488 characters (Fig. 2). The *Scheffersomyces* clade was divided into three subclades: 1) the early diverging *S. spartinae* and *S. gosingicus* subclade, 2) the cellobiose-fermenting *S. ergatensis* subclade, and 3) the largest, xylose-fermenting *S. stipitis* subclade to which the three new species belong.

Addition of more taxa and more molecular data in phylogenetic analyses has helped to define monophyletic clades, among a number of genera now recognized as polyphyletic. One problematic taxon, *Pichia*, was based primarily on ascospore shape. Hat-shaped ascospores, however, are found among several distant clades of yeasts as well as distantly related members of the Pezizomycotina. *Scheffersomyces* was proposed recently for species in the *Pichia stipitis* clade [55]. The genus included the type species, *S. stipitis*, and *S. ergatensis* and *S. spartinae* [55]. We propose additional new combinations in the genus *Scheffersomyces* by including clade members that previously were described as asexual species of the polyphyletic genus *Candida* [77].

1. ***Scheffersomyces coipomoensis*** (C. Ramírez & A. González) H. Urbina & M. Blackw. comb. nov.

MycoBank accession number: MB563714.

Basionym: *Candida coipomoensis* C. Ramírez & A. González, Mycopathologia 88:84, 1984.

1. ***Scheffersomyces gosingicus*** (C.F. Lee) H. Urbina & M. Blackw. comb. nov.

MycoBank accession number: MB563799.

Basionym: *Candida gosingica* C.F. Lee, Int J Syst Evol Microbiol 61:670, 2011.

1. ***Scheffersomyces insectosa*** (Kurtzman) H. Urbina & M. Blackw. comb. nov.

MycoBank accession number: MB 564799.

Basionym: *Candida shehatae* var. *insectosa* Kurtzman, Antonie van Leeuwenhoek 57:218, 1990. Synonym: *Candida insectosa* (Kurtzman) Kurtzman, in Kurtzman & M. Suzuki, Mycoscience 51:10, 2010.

1. ***Scheffersomyces lignicola*** (Jindam., Limtong, Yongman., Tuntir., Potach., H. Kawas & Nakase) H. Urbina & M. Blackw. comb. nov.

MycoBank accession number: MB 563803.

Basionym: *Candida lignicola* Jindam., Limtong, Yongman., Tuntir., Potach., H. Kawas & Nakase, FEMS Yeast Res 7:1412, 2007.

1. ***Scheffersomyces lignosus*** (Kurtzman) H. Urbina & M. Blackw. comb. nov.

MycoBank accession number: MB 563801.

Basionym: *Candida shehatae* var. *lignosa* Kurtzman, Antonie van Leeuwenhoek 57:218, 1990. Synonym: *Candida lignosa* (Kurtzman) Kurtzman, in Kurtzman & M. Suzuki, Mycoscience 51:10, 2010.

1. ***Scheffersomyces queiroziae*** (R.O. Santos, R.M. Cadete, Badotti, A. Mouro, Wallheim, F.C.O. Gomes, Stambuk, Lachance & C.A. Rosa) H. Urbina & M. Blackw. comb. nov.

MycoBank accession number: MB563717.

Basionym: *Candida queiroziae* R.O. Santos, R.M. Cadete, Badotti, A. Mouro, Wallheim, F.C.O. Gomes, Stambuk, Lachance & C.A. Rosa, Antonie van Leeuwenhoek 99:639. 2011.

1. ***Scheffersomyces shehatae*** (H.R. Buckley & van Uden) H. Urbina & M. Blackw. comb. nov.

MycoBank accession number: MB563716.

Basionym: *Candida shehatae* H.R. Buckley & van Uden, Mycopathologia Mycol. appl. 34:297, 1966.

Xylose reductase among X-F yeast members of the S. stipitis subclade

We amplified a ∼600 bp PCR product of *XYL1* from X-F and non X-F yeasts tested (Fig. 5). The translated protein sequences at the N-terminal region have the conserved amino acids 49-D, 51-A, and 54-Y, described as part of the catalytic GX_3_DXAX_2_Y domain; the LX_8_DX_4_H and the GX_3_GXG domains, and the amino acids 83-K, 132-P, and 167-K that form the xylose-binding pocket previously reported [78], [79] (Fig. 6). Conserved amino acid substitutions at positions 64, 170, 194 (I  =  V); 65 (N  =  K); 69 (K  =  D); 70 (D  =  E); 80, 178 (I  =  L); 107 (L  =  V); 127 (N  =  E); 149 (L  =  I), 150 (A  =  L), 174 (T  =  P), 179 (Y  =  L), 184 (S  =  G), 186 (E  =  T), 188 (P  =  K), and 204 (S  =  P) and the percentage of conserved substitutions usually were higher than 50% at all sites compared with the non X-F fermenting yeast *S. ergatensis* (Fig. 6).

## Discussion

### Yeasts Sampled from Rotted Wood

Most of the isolates from live oak and water oak were members of the *Sugiyamaella* clade: *Candida boreocaroliniensis*, *Candida lignohabitans*, and *S. smithiae*. The cosmopolitan genus *Sugiyamaella* is comprised of yeasts reported primarily from wood and frass of lignicolous beetles. It is of interest that, unlike other yeasts from these habitats, they are unable to ferment D-xylose [80], [81]. We also isolated other non X-F species *Candida athensensis, Trichomonascus petasosporus*, and a close relative of *Candida anneliseae*, ascomycete yeasts that previously were found associated with fungus-feeding beetles collected in Panama and the USA [56].

The yeast strains isolated from pecan wood, on the contrary, were dominated by the new X-F yeast species, *S. illinoinensis*, a close relative of *S. stipitis.* Only this wood was inhabited by *Odontotaenius disjunctus* (Passalidae, Coleoptera) in our study. Zhang *et al.* [82] recognized the relationship between *S. stipitis* and the lignicolous beetle commonly found inhabiting decayed hardwoods in the southeastern US, so the phylogenetic placement of these closely related species is consistent with the previous findings.

Other members of the *Scheffersomyces* clade (*S. ergatensis, S. shehatae,* and *S. stipitis*) have been reported frequently from associations with the gut of lignicolous beetles, including, not only the passalid beetle *O. disjunctus* but also beetles in the families Cerambycidae, Lucanidae, Buprestidiae, and Tenebrionidae [22], [83]. It is likely that the common gut yeasts are efficient at digesting components of the host diet, resisting toxic secondary metabolites, and adapting to the gut physiological environment (low oxygen and high carbon dioxide concentrations and extreme pH variation), characteristics that give them a greater chance to be horizontally transmitted to progeny. Consequently, in each host generation the symbiotic yeasts may be exposed to bottlenecks and positive selection driven by the host beetles, and these selective pressures increase when changes in the host diet occur. These evolutionary processes could have favored rapid speciation with morphological and other traditional characters lagging behind molecular changes in the *Scheffersomyces* yeast members. Evidence that supports our hypothesis is: 1) *S. shehatae, S. lignicola*, and *S. insectosa*, often found in association with insects, are indistinguishable by morphology and some molecular markers (e.g. SSU and D1/D2 LSU); 2) branch lengths are constrained in the phylogenetic tree (Fig. 2); and 3) gut morphology is modified to enhance the horizontal transmission of gut yeasts across generations, e.g. the posterior hindgut region of *O. disjunctus* is colonized mainly by filamentous yeasts attached by a holdfast [84]; in addition mycetomes occur in lignicolous cerambycid beetles colonized exclusively by closely related species of *S. shehatae* [22].

Kurtzman [58] recognized *Candida shehatae* var. *shehatae*, var. *insectosa*, and var. *lignicola* based on biochemical assays, a single nucleotide difference in the D1/D2 LSU region, and identical SSU rRNA among the varieties. He suggested that in order to understand the phylogenetic relationship between varieties of *C. shehatae*, analyses including ITS rRNA should be included. More recently, these varieties were raised to species level based on their distinctive electrokariotype profiles and the reinterpretation of the D1/D2 LSU locus [55], [57]. Yeast species often have been underestimated on the basis of only LSU and SSU rRNA data [85]-[87]. Therefore, the addition of more molecular markers in phylogenetic analyses has been used to increase the power of species recognition (see next section).

### Phylogenetic Study of the *Scheffersomyces* Clade

Lachance *et al.* [88] proposed a method for species delimitation in yeasts based on parsimony networks. In our experience, the results obtained by using this method are difficult to interpret for several reasons: 1) the species are plotted as isolated entities with little information on phylogenetic relationship among members; 2) the method does not allow inclusion of a model of nucleotide selection for the analysis; and 3) node support values are lacking.

Because of these disadvantages, we implemented, instead, a multilocus phylogenetic analysis based on ML, commonly applied to fungi. We incorporated additional loci (ITS and *RPB1*) in order to increase the robustness of the phylogenetic analysis in the study of the *Scheffersomyces* members. We followed the recommendations of the Assembling the Fungal Tree of Life (AFTOL http://aftol.org/) research group in searching for orthologous genes in fungal genomes [89]. In addition results obtained by Schoch *et al.* [90] showed that *RPB1*, *RPB2*, and *TEF1* (elongation factor 1 alpha) are more phylogenetically informative compared to rRNA genes in Ascomycota. More recently, Schoch *et al.* [59] proposed the use of ITS as a barcode gene for fungi, although *RPB1* had higher species discriminatory power than ITS in Saccharomycotina. The authors [59] also pointed out that datasets containing combinations of at least three molecular markers (SSU, LSU, ITS or *RPB1*) showed the highest probability of correct identification for all Fungi.

As mentioned in the results section, the *Scheffersomyces* clade, including all known taxa reported in the literature, is comprised of three subclades: 1) an early diverged subclade of *S. spartinae,* the only member that lacks the ability to ferment both xylose and cellobiose, and *S. gosingicus,* a cellobiose-fermenting (C-F) yeast isolated from soil in southeastern Asia [91]; 2) a C-F subclade that includes *Scheffersomyces* sp. UFMG-HMD-26.3 (not yet validly published [20]), *S. coipomoensis, S. ergatensis, S. lignicola,* and *S. queiroziae*; and 3) the X-F subclade containing *S. lignosus, S. illinoinensis, S. insectosa, S. quercinus, S. segobiensis, S. stipitis, S. shehatae*, and *S. virginianus* (Fig. 2).

The phylogenetic outcome also suggests that the common ancestor of the *Scheffersomyces* clade may have shown the ability to ferment D-xylose and cellobiose. This hypothesis is supported by the phylogenetic analyses based on a multilocus dataset that places *S. gosingicus,* a C-F species, in the earliest derived subclade, and the results of the phylogenetic study showing XR presence in all *Scheffersomyces* clade members studied (Fig. 5). Moreover, the same tree topology of the multilocus analysis was recovered by using either *XYL1* or XR, suggesting that X-F ability might have played a fundamental role in the speciation process of the X-F subclade (Figs. 2, 5). We did not include the single copy gene *XYL1* in the multilocus phylogenetic analysis because the orthology of this locus has not been confirmed across several yeast taxa.

The ability to ferment both wood components (D-xylose and cellobiose) is exhibited by only a few yeasts: *Ogataea polymorpha* [92], *Brettanomyces naardenensis* [1], and *Spathaspora passalidarum* [18], and in these species fermentative abilities are weak or delayed. On the contrary, *Scheffersomyces* clade members exhibit only one or the other fermentative ability. The loss of fermentation capability could be a consequence of becoming more efficient in carrying out the fermentation of fewer sugars. In particular, the fermentation of cellobiose has an antagonistic effect against the fermentation of D-xylose, because during the extracellular fermentation of cellobiose, units of glucose are released by β-glucosidase, a sugar that has higher affinity for the pentose membrane transporter rather than D-xylose. Consequently, glucose is first incorporated into the cells to be fermented [93].

### XR in the *Scheffersomyces* Clade Members

The metabolic constraint in the regeneration of the cofactor NAD^+^ to NADH^+^ has been described as a major constraint in the fermentation of D-xylose, therefore most studies have focused on the molecular characterization of the conserved domains involved in the uptake of this cofactor that is present in the C-terminal region of the XR, but few studies have been done on the characterization of the N-terminal region.

In the study in the N-terminal region of the XR of the X-F members of the *S. stipitis* subclade we were able to identify all of the conserved domains and amino acids in both X-F and non X-F yeasts. These findings indicated that the ability to ferment xylose does not rely solely on the presence of these conserved regions. We also found several biased nucleotide mutations that maintain the same polarity as the codified amino acid, and only the mutations on the residues 174, 179, 184, 188, and 204 showed a change in amino acid polarity. These mutations were found mainly surrounding conserved domains in comparison with the amino acid sequence of the non X-F yeast *S. ergatensis* (Fig. 6). The mutations could generate structural modifications that allow the fermentation of xylose in the *Scheffersomyces* clade members, results that could be supported by performing direct mutagenesis studies on the amino acids to characterize their individual roles in the performance of XR.

Although the X-F yeasts were dispersed throughout the Saccharomycotina, as we mentioned in the introduction, the Debaryomycetaceae includes the largest number of X-F yeasts, such as species of *Scheffersomyces* and *Spathaspora* found in association with lignicolous insects [21], [24]. This phylogenetic placement of the X-F yeasts supports two alternatives: the X-F ability was the result of convergent evolution in ascomycete yeasts, or the X-F ability was present in the earliest common ancestor in the Saccharomycotina and has been retained mainly in the yeasts associated with lignicolous habitats. Several independent lines of evidence favor the second premise: 1) classical biochemical studies have determined that the ability to assimilate D-xylose and xylitol is common throughout the yeasts; 2) several relatively early diverging yeasts, *B. naardenensis*, *O. polymorpha,* and *P. tannophilus*, ferment xylose [1], [92]; 3) many studies characterizing yeast diversity indicate that most X-F yeasts are associated with wood and the gut of lignicolous insects [21]; and 4) more recently, a report of the genome sequences of a diverse group of X-F and non X-F yeasts confirmed the presence of xylose genes in all of them [45].

Yeasts as a group, are known for their biochemical versatility in utilizing a wide variety of carbon sources. Individual strains, however, may be characterized by specific physiological profiles depending on their life style and environment. Wood substrates and the gut of lignicolous insects previously were unexplored environments for the isolation of X-F yeasts. The findings of this study further support the hypothesis that X-F yeasts and yeasts in the *Sugiyamaella* clade are common inhabitants of the wood substrates. The *Scheffersomyces* clade is comprised mainly of cellobiose- and D-xylose-fermenting yeasts isolated from distant geographical regions and associated with wood and insects that feed on plant tissues. The amino acid modifications present in the putative XR of X-F yeasts in the *S. stipitis* subclade, could be responsible for the enhanced rate of fermentation shown by the members of this clade. The addition of ITS and *RPB1* loci in the phylogenetic studies on the *Scheffersomyces* clade dramatically increased the support of the phylogenetic relationships of the members. We have used the primers for *XYL1* designed for this study across several yeast species (data not show), and they could be used to help understand how these genes have evolved in the members of Saccharomycotina. The phylogenetic reconstruction using only *XYL1* or *RPB1* was similar to the multilocus analysis, and these loci have potential for rapid identification of cryptic species in this clade.

## References

[1] Kurtzman CP, Fell JW, Boekhout T, editors (2011). The yeasts, a taxonomy study..

[2] Jeffries TW, Kurtzman CP (1994). Strain selection, taxonomy, and genetics of close-fermenting yeasts.. Enzyme Microb Tech.

[3] Hahn-Hagerdal B, Karhumaa K, Jeppsson M, Gorwa-Grauslund MF (2007). Metabolic engineering for pentose utilization in *Saccharomyces cerevisiae*.. Adv Biochem Eng Biotechnol.

[4] Hahn-Hagerdal B, Karhumaa K, Fonseca C, Spencer-Martins I, Gorwa-Grauslund MF (2007). Towards industrial pentose-fermenting yeast strains.. Appl Microbiol Biotechnol.

[5] Jeffries TW, Shi NQ (1999). Genetic engineering for improved xylose fermentation by yeasts.. Adv Biochem Eng Biotechnol.

[6] Shi NQ, Davis B, Sherman F, Cruz J, Jeffries TW (1999). Disruption of the cytochrome c gene in xylose-utilizing yeast *Pichia stipitis* leads to higher ethanol production.. Yeast.

[7] Hamacher T, Becker J, Gardonyi M, Hahn-Hagerdal B, Boles E (2002). Characterization of the xylose-transporting properties of yeast hexose transporters and their influence on xylose utilization.. Microbiology.

[8] Jin YS, Ni H, Laplaza JM, Jeffries TW (2003). Optimal growth and ethanol production from xylose by recombinant *Saccharomyces cerevisiae* require moderate D-xylulokinase activity.. Appl Environ Microbiol.

[9] Jeffries TW, Grigoriev IV, Grimwood J, Laplaza JM, Aerts A (2007). Genome sequence of the lignocellulose-bioconverting and xylose-fermenting yeast *Pichia stipitis*.. Nat Biotechnol.

[10] Karhumaa K, Sanchez RG, Hahn-Hagerdal B, Gorwa-Grauslund MF (2007). Comparison of the xylose reductase-xylitol dehydrogenase and the xylose isomerase pathways for xylose fermentation by recombinant *Saccharomyces cerevisiae*.. Microb Cell Fact.

[11] Karhumaa K, Fromanger R, Hahn-Hagerdal B, Gorwa-Grauslund MF (2007). High activity of xylose reductase and xylitol dehydrogenase improves xylose fermentation by recombinant *Saccharomyces cerevisiae*.. Appl Microbiol Biotechnol.

[12] Hughes SR, Sterner DE, Bischoff KM, Hector RE, Dowd PF (2009). Engineered *Saccharomyces cerevisiae* strain for improved xylose utilization with a three-plasmid SUMO yeast expression system.. Plasmid.

[13] Hector RE, Qureshi N, Hughes SR, Cotta MA (2008). Expression of a heterologous xylose transporter in a *Saccharomyces cerevisiae* strain engineered to utilize xylose improves aerobic xylose consumption.. Appl Microbiol Biotechnol.

[14] Kumar S, Gummadi SN (2011). Metabolism of glucose and xylose as single and mixed feed in *Debaryomyces nepalensis* NCYC 3413: production of industrially important metabolites.. Appl Microbiol Biotechnol.

[15] Shi NQ, Cruz J, Sherman F, Jeffries TW (2002). SHAM-sensitive alternative respiration in the xylose-metabolizing yeast *Pichia stipitis*.. Yeast.

[16] Jeffries TW (2006). Engineering yeasts for xylose metabolism.. Curr Opin Biotechnol.

[17] Van Vleet JH, Jeffries TW, Olsson L (2008). Deleting the para-nitrophenyl phosphatase (pNPPase), PHO13, in recombinant *Saccharomyces cerevisiae* improves growth and ethanol production on D-xylose.. Metab Eng.

[18] Nguyen NH, Suh SO, Marshall CJ, Blackwell M (2006). Morphological and ecological similarities: wood-boring beetles associated with novel xylose-fermenting yeasts, *Spathaspora passalidarum* gen. sp. nov. and *Candida jeffriesii* sp. nov.. Mycol Res.

[19] Cadete RM, Santos RO, Melo MA, Mouro A, Goncalves DL (2009). *Spathaspora arborariae* sp. nov., a D-xylose-fermenting yeast species isolated from rotting wood in Brazil.. FEMS Yeast Res.

[20] Cadete RM, Melo MA, Lopes MR, Pereira GM (2011). *Candida amazonensis* sp.. nov., an ascomycetous yeast isolated from rotting wood in Amazonian Forest, Brazil. Int J Syst Evol Microbiol.

[21] Suh SO, Marshall CJ, McHugh JV, Blackwell M (2003). Wood ingestion by passalid beetles in the presence of xylose-fermenting gut yeasts.. Mol Ecol.

[22] Grunwald S, Pilhofer M, Holl W (2010). Microbial associations in gut systems of wood- and bark-inhabiting longhorned beetles [Coleoptera: Cerambycidae].. Syst Appl Microbiol.

[23] Tanahashi M, Kubota K, Matsushita N, Togashi K (2010). Discovery of mycangia and the associated xylose-fermenting yeasts in stag beetles (Coleoptera: Lucanidae).. Naturwissenschaften.

[24] Suh SO, Blackwell M, Kurtzman CP, Lachance MA (2006). Phylogenetics of Saccharomycetales, the ascomycete yeasts.. Mycologia.

[25] Toivola A, Yarrow D, van den Bosch E, van Dijken JP, Scheffers WA (1984). Alcoholic fermentation of D-xylose by yeasts.. Appl Environ Microbiol.

[26] Delgenes JP, Moletta R, Navarro JM (1989). Fermentation of D-xylose, D-glucose, L-arabinose mixture by *Pichia stipitis*: Effect of the oxygen transfer rate on fermentation performance.. Biotechnol Bioeng.

[27] Delgenes JP, Moletta R, Navarro JM (1986). The effect of aeration on D-xylose fermentation by *Pachysolen tannophilus*, *Pichia stipitis*, *Kluyveromyces marxianus* and *Candida shehatae*.. Biotechnol Lett.

[28] Du Preez JC, Bosch M, Prior BA (1986). Xylose fermentation by *Candida shehatae* and *Pichia stipitis*, effects of pH, temperature and substrate concentration.. Enzyme Microb Tech.

[29] Parekh SR, Yu S, Wayman M (1986). Adaptation of *Candida shehatae* and *Pichia stipitis* to wood hydrolysates for increased ethanol production.. Appl Microbiol Biotechnol.

[30] Parekh S, Wayman M (1986). Fermentation of cellobiose and wood sugars to ethanol by *Candida shehatae* and *Pichia stipitis*.. Biotechnol Lett.

[31] Sreenath HK, Chapman TW, Jeffries TW (1986). Ethanol production from D-xylose in batch fermentations with *Candida shehatae*: Process variables.. Appl Microbiol Biotechnol.

[32] Alexander MA, Chapman TW, Jeffries TW (1988). Xylose metabolism by *Candida shehatae* in continuous culture.. Appl Microbiol Biotechnol.

[33] Alexander MA, Chapman TW, Jeffries TW (1987). Continuous ethanol production from D-xylose by *Candida shehatae*.. Biotechnol Bioeng.

[34] Prior BA, Alexander MA, Yang V, Jeffries TW (1988). The role of alcohol-dehydrogenase in the fermentation of D-xylose by *Candida shehatae* ATCC-22984.. Biotechnol Lett.

[35] Ho NW, Lin FP, Huang S, Andrews PC, Tsao GT (1990). Purification, characterization, and amino terminal sequence of xylose reductase from *Candida shehatae*.. Enzyme Microb Tech.

[36] Palnitkar S, Lachke A (1992). Effect of nitrogen sources on oxidoreductive enzymes and ethanol production during D-xylose fermentation by *Candida shehatae*.. Can J Microbiol.

[37] Palnitkar SS, Lachke AH (1990). Efficient simultaneous saccharification and fermentation of agricultural residues by *Saccharomyces cerevisiae* and *Candida shehatae*. The D-xylose fermenting yeast.. Appl Biochem Biotechnol.

[38] Agbogbo FK, Wenger KS (2007). Production of ethanol from corn stover hemicellulose hydrolyzate using *Pichia stipitis*.. J Ind Microbiol Biotechnol.

[39] Agbogbo FK, Coward-Kelly G (2008). Cellulosic ethanol production using the naturally occurring xylose-fermenting yeast, *Pichia stipitis*.. Biotechnol Lett.

[40] Agbogbo FK, Haagensen FD, Milam D, Wenger KS (2008). Fermentation of acid-pretreated corn stover to ethanol without detoxification using *Pichia stipitis*.. Appl Biochem Biotechnol.

[41] Agbogbo FK, Wenger KS (2006). Effect of pretreatment chemicals on xylose fermentation by *Pichia stipitis*.. Biotechnol Lett.

[42] Jeffries TW (1986). Regulation of the xylose fermentation in *Candida shehatae* and *Pachysolen tannophilus*.. Abstr Pap Am Chem S.

[43] Jeffries TW, Van VleetJR (2009). *Pichia stipitis* genomics, transcriptomics, and gene clusters.. FEMS Yeast Res.

[44] Lee JW, Zhu JY, Scordia D, Jeffries TW (2011). Evaluation of ethanol production from corncob using *Scheffersomyces* (*Pichia*) *stipitis* CBS 6054 by volumetric scale-up.. Appl Biochem Biotechnol.

[45] Wohlbach DJ, Kuo A, Sato TK, Potts KM, Salamov AA (2011). Comparative genomics of xylose-fermenting fungi for enhanced biofuel production.. Proc Natl Acad Sci U S A.

[46] Jeppsson M, Bengtsson O, Franke K, Lee H, Hahn-Hagerdal B (2006). The expression of a *Pichia stipitis* xylose reductase mutant with higher K(M) for NADPH increases ethanol production from xylose in recombinant *Saccharomyces cerevisiae*.. Biotechnol Bioeng.

[47] Fromanger R, Guillouet SE, Uribelarrea JL, Molina-Jouve C, Cameleyre X (2010). Effect of controlled oxygen limitation on *Candida shehatae* physiology for ethanol production from xylose and glucose.. J Ind Microbiol Biotechnol.

[48] Zhang J, Yang M, Tian S, Zhang Y, Yang X (2010). Co-expression of xylose reductase gene from *Candida shehatae* and endogenous xylitol dehydrogenase gene in *Saccharomyces cerevisiae* and the effect of metabolizing xylose to ethanol.. Prikl Biokhim Mikrobiol.

[49] Bajwa PK, Phaenark C, Grant N, Zhang X, Paice M (2011). Ethanol production from selected lignocellulosic hydrolysates by genome shuffled strains of *Scheffersomyces stipitis*.. Bioresour Technol.

[50] Khattab SM, Watanabe S, Saimura M, Kodaki T (2011). A novel strictly NADPH-dependent *Pichia stipitis* xylose reductase constructed by site-directed mutagenesis.. Biochem Biophys Res Commun.

[51] Hughes SR, Gibbons WR, Bang SS, Pinkelman R, Bischoff KM (2012). Random UV-C mutagenesis of *Scheffersomyces* (formerly *Pichia*) *stipitis* NRRL Y-7124 to improve anaerobic growth on lignocellulosic sugars.. J Ind Microbiol Biotechnol.

[52] Kreger-van Rij NJW, Lodder J (1970). *Pichia* Hansen..

[53] Vaughan-Martini A (1984). Comparazione dei genomi del lievito *Pichia stipitis* e di alcune specie imperfette affini. Ann. Fac. Agrar. Univ.. Perugia.

[54] Kurtzman CP, Robnett CJ (1998). Identification and phylogeny of ascomycetous yeasts from analysis of nuclear large subunit (26S) ribosomal DNA partial sequences.. Antonie Van Leeuwenhoek.

[55] Kurtzman CP, Suzuki M (2010). Phylogenetic analysis of ascomycete yeasts that form coenzyme Q-9 and the proposal of the new genera *Babjeviella*, *Meyerozyma*, *Millerozyma*, *Priceomyces*, and *Scheffersomyces*.. Mycoscience.

[56] Suh SO, White MM, Nguyen NH, Blackwell M (2004). The status and characterization of Enteroramus dimorphus: a xylose-fermenting yeast attached to the gut of beetles.. Mycologia.

[57] Passoth V, Hansen M, Klinner U, Emeis CC (1992). The electrophoretic banding pattern of the chromosomes of *Pichia stipitis* and *Candida shehatae*.. Curr Genet.

[58] Kurtzman CP (1990). *Candida shehatae* genetic diversity and phylogenetic relationships with other xylose-fermenting yeasts.. Antonie Van Leeuwenhoek.

[59] Schoch CL, Seifert KA, Huhndorf S, Robert V, Spouge JL, Levesque CA, Chen W, the Barcoding Consortium (2012). The internal transcribed spacer as a universal DNA barcode marker for Fungi. Fungal Barcoding Consortium.. Proc Natl Acad Sci U S A.

[60] Suh SO, McHugh JV, Blackwell M (2004). Expansion of the *Candida tanzawaensis* yeast clade: 16 novel *Candida* species from basidiocarp-feeding beetles.. Int J Syst Evol Microbiol.

[61] White TJ, Bruns TD, Lee S, Taylor JW, Innis MA, Gelfand DH, Sninsky JJ, White TJ (1990). Amplification and direct sequencing of fungal ribosomal RNA genes for phylogenetics..

[62] Hibbett DS (1996). Phylogenetic evidence for horizontal transmission of group I introns in the nuclear ribosomal DNA of mushroom-forming fungi.. Mol Biol Evol.

[63] Matheny PB, Liu YJJ, Ammirati JF, Hall BD (2002). Using RPB1 sequences to improve phylogenetic inference among mushrooms (*Inocybe*, Agaricales).. Am J Bot.

[64] Tanabe Y, Saikawa M, Watanabe MM, Sugiyama J (2004). Molecular phylogeny of Zygomycota based on *EF-1* alpha and *RPB1* sequences: limitations and utility of alternative markers to rDNA.. Mol Phylogenet Evol.

[65] Sullivan DJ, Westerneng TJ, Haynes KA, Bennett DE, Coleman DC (1995). *Candida dubliniensis* sp. nov.: phenotypic and molecular characterization of a novel species associated with oral candidosis in HIV-infected individuals.. Microbiology 141 (Pt.

[66] Fadda ME, Mossa V, Pisano MB, Deplano M, Cosentino S (2004). Occurrence and characterization of yeasts isolated from artisanal Fiore Sardo cheese.. Int J Food Microbiol.

[67] Fadda ME, Viale S, Deplano M, Pisano MB, Cosentino S (2010). Characterization of yeast population and molecular fingerprinting of *Candida zeylanoides* isolated from goat's milk collected in Sardinia.. Int J Food Microbiol.

[68] Fuentefria AM, Suh SO, Landell MF, Faganello J, Schrank A (2008). *Trichosporon insectorum* sp. nov., a new anamorphic basidiomycetous killer yeast.. Mycol Res.

[69] Yarrow D, Kurtzman CP, Fell JW (1998). Methods for the isolation, maintenance and identification of yeasts..

[70] Barnett JA, Payne RW, Yarrow D (2000). Yeasts: Characteristics and identification. Cambridge: Cambridge University Press.. x+1139 p.

[71] Maddison W, Maddison D (2005). Mesquite: A modular system for evolutionary analysis.. Evolution.

[72] Liu K, Warnow TJ, Holder MT, Nelesen SM, Yu J (2012). SATe-II: Very fast and accurate simultaneous estimation of multiple sequence alignments and phylogenetic trees.. Syst Biol.

[73] Stamatakis A (2006). RAxML-VI-HPC: maximum likelihood-based phylogenetic analyses with thousands of taxa and mixed models.. Bioinformatics.

[74] Ohama T, Suzuki T, Mori M, Osawa S, Ueda T (1993). Non-universal decoding of the leucine codon CUG in several *Candida* species.. Nucleic Acids Res.

[75] Muller T, Vingron M (2000). Modeling amino acid replacement.. J Comput Biol.

[76] Milan EP, Kallas EG, Costa PR, da Matta DA, Lopes Colombo A (2001). Oral colonization by *Candida* spp. among AIDS household contacts.. Mycoses.

[77] Knapp S, McNeill J, Turland NJ (2011). Changes to publication requirements made at the XVIII International Botanical Congress in Melbourne: What does e-publication mean for you?. Taxon.

[78] Chu BC, Lee H (2006). Investigation of the role of a conserved glycine motif in the *Saccharomyces cerevisiae* xylose reductase.. Curr Microbiol.

[79] Zhang Q, Shirley NJ, Burton RA, Lahnstein J, Hrmova M (2010). The genetics, transcriptional profiles, and catalytic properties of UDP-alpha-D-xylose 4-epimerases from barley.. Plant Physiol.

[80] Kurtzman CP, Robnett CJ (2007). Multigene phylogenetic analysis of the *Trichomonascus*, *Wickerhamiella* and *Zygoascus* yeast clades, and the proposal of *Sugiyamaella* gen. nov. and 14 new species combinations.. FEMS Yeast Res.

[81] Houseknecht JL, Hart EL, Suh SO, Zhou JJ (2011). Yeasts in the *Sugiyamaella* clade associated with wood-ingesting beetles and the proposal of *Candida bullrunensis* sp. nov.. Int J Syst Evol Microbiol.

[82] Zhang N, Suh SO, Blackwell M (2003). Microorganisms in the gut of beetles: evidence from molecular cloning.. J Invertebr Pathol.

[83] Gaster PF, Rosa CA, Gabor P (2006). Yeast and invertebrate associations..

[84] Nardi JB, Bee CM, Miller LA, Nguyen NH, Suh SO (2006). Communities of microbes that inhabit the changing hindgut landscape of a subsocial beetle.. Arthropod Struct Dev.

[85] Rokas A, Williams BL, King N, Carroll SB (2003). Genome-scale approaches to resolving incongruence in molecular phylogenies.. Nature.

[86] Cadez N, Raspor P, Smith MT (2006). Phylogenetic placement of *Hanseniaspora*-*Kloeckera* species using multigene sequence analysis with taxonomic implications: descriptions of *Hanseniaspora pseudoguilliermondii* sp. nov. and *Hanseniaspora occidentalis* var. *citrica* var. nov.. Int J Syst Evol Microbiol.

[87] Liti G, Barton DB, Louis EJ (2006). Sequence diversity, reproductive isolation and species concepts in *Saccharomyces*.. Genetics.

[88] Lachance MA, Dobson J, Wijayanayaka DN, Smith AM (2010). The use of parsimony network analysis for the formal delineation of phylogenetic species of yeasts: *Candida apicola, Candida azyma,* and *Candida parazyma* sp. nov., cosmopolitan yeasts associated with floricolous insects.. Antonie Van Leeuwenhoek.

[89] Robbertse B, Reeves JB, Schoch CL, Spatafora JW (2006). A phylogenomic analysis of the Ascomycota.. Fungal Genet Biol.

[90] Schoch CL, Sung GH, Lopez-Giraldez F, Townsend JP, Miadlikowska J (2009). The Ascomycota tree of life: a phylum-wide phylogeny clarifies the origin and evolution of fundamental reproductive and ecological traits.. Syst Biol.

[91] Chang CF, Yao CH, Young SS, Limtong S, Kaewwichian R (2011). *Candida gosingica* sp. nov., an anamorphic ascomycetous yeast closely related to *Scheffersomyces spartinae*.. Int J Syst Evol Microbiol.

[92] Ryabova OB, Chmil OM, Sibirny AA (2003). Xylose and cellobiose fermentation to ethanol by the thermotolerant methylotrophic yeast *Hansenula polymorpha*.. FEMS Yeast Res.

[93] Han JH, Park JY, Yoo KS, Kang HW, Choi GW (2011). Effect of glucose on xylose utilization in *Saccharomyces cerevisiae* harboring the xylose reductase gene.. Arch Microbiol.

